# Integrating a Virtual ICU with Cardiac and Cardiovascular ICUs: Managing the Needs of a Complex and High-Acuity Specialty ICU Cohort

**DOI:** 10.14797/mdcvj.1247

**Published:** 2023-08-01

**Authors:** Atiya Dhala, Mario V. Fusaro, Faisal Uddin, Divina Tuazon, Steven Klahn, Roberta Schwartz, Farzan Sasangohar, Jefferson Alegria, Faisal Masud

**Affiliations:** 1 Houston Methodist Hospital, Houston, Texas, US; 2 Equum Medical, New York, New York, US; 3 Houston Methodist DeBakey Heart & Vascular Center, Houston Methodist Hospital, Houston, Texas, US; 4 Department of Virtual Medicine, Houston Methodist Hospital, Houston, Texas, US; 5 Houston Methodist Academic Institute, Houston Methodist Hospital, Houston, Texas, US; 6 Texas A&M University, College Station, Texas, US

**Keywords:** critical care, tele-critical care, cardiac ICU, cardiovascular ICU, cardiothoracic ICU, staffing, burnout, patient outcomes, predictive medicine

## Abstract

A long-standing shortage of critical care intensivists and nurses, exacerbated by the coronavirus disease (COVID-19) pandemic, has led to an accelerated adoption of tele-critical care in the United States (US). Due to their complex and high-acuity nature, cardiac, cardiovascular, and cardiothoracic intensive care units (ICUs) have generally been limited in their ability to leverage tele-critical care resources. In early 2020, Houston Methodist Hospital (HMH) launched its tele-critical care program called Virtual ICU, or vICU, to improve its ICU staffing efficiency while providing high-quality, continuous access to in-person and virtual intensivists and critical care nurses. This article provides a roadmap with prescriptive specifications for planning, launching, and integrating vICU services within cardiac and cardiovascular ICUs—one of the first such integrations among the leading academic US hospitals. The success of integrating vICU depends upon the (1) recruitment of intensivists and RNs with expertise in managing cardiac and cardiovascular patients on the vICU staff as well as concerted efforts to promote mutual trust and confidence between in-person and virtual providers, (2) consultations with the bedside clinicians to secure their buy-in on the merits of vICU resources, and (3) collaborative approaches to improve workflow protocols and communications. Integration of vICU has resulted in the reduction of monthly night-call requirements for the in-person intensivists and an increase in work satisfaction. Data also show that support of the vICU is associated with a significant reduction in the rate of Code Blue events (denoting a situation where a patient requires immediate resuscitation, typically due to a cardiac or respiratory arrest). As the providers become more comfortable with the advances in artificial intelligence and big data-driven technology, the Cardiac ICU Cohort continues to improve methods to predict and track patient trends in the ICUs.

## Introduction

Historians of the early 21st century will likely identify the SARS-CoV-2 (COVID-19) pandemic as the key inflection point when remote delivery of services across disparate industries became not only normative but essential.^1,2^ While tele-critical care or the virtual intensive care unit (vICU) expanded patient access to critical care medicine decades ago, the COVID-19 pandemic accelerated the pace of vICU adoption.^3^ Even before the pandemic, hospitals with staffing models that aim to provide 24/7 in-person bedside critical care expertise often suffered from significant cost and utility inefficiencies while also contributing to higher burnout rates for critical care clinicians.^4,5,6^ For these institutions, vICU represents a technology-based forward-looking solution that addresses the current need for improved staffing and scalability while holding the promise of better patient outcomes using predictive medicine and augmented intelligence.

Houston Methodist Hospital (HMH) in the Texas Medical Center and its seven regional hospitals serving the Greater Houston area (collectively “HM”) had been exploring the feasibility of a vICU program well before the outbreak of COVID-19 in early 2020.^7^ The new vICU program under consideration offered a staffing solution that initially would provide improved nocturnal support to bedside ICU clinicians using critical care virtual MDs (vMDs) and virtual critical care RNs (vRNs) from a centralized operations center, thereby reducing both the need for locums staffing and the workload of bedside clinicians. Over time, the vICU would expand to include 24-hour support, remove staffing inefficiencies, and maintain patient care and safety levels while optimizing the cost structure for the delivery of critical care services. As COVID-19 surged, HMH fast-tracked the rollout of the vICU to all of HM’s 15 ICUs.^8^

As part of HM’s globally recognized Methodist DeBakey Heart & Vascular Center, the cardiac ICU (CICU, 30 beds) and the cardiovascular ICU (CVICU, 36 beds) together constitute the highly specialized Cardiac ICU Cohort. The unique demands of patient care in the Cardiac ICU Cohort have generally limited the hospital’s ability to provide tele-critical care to these ICUs. Consequently, tele-critical care had not been widely adopted to serve cardiac ICUs by many of the leading US academic medical institutions. Without any known roadmap for a successful integration of vICU with cardiac ICUs, HMH found itself in unchartered territory. This article aims to provide a roadmap with prescriptive specification about the special requirements of patient care in the CICU and CVICU, the framework of integrating a vICU into the Cardiac ICU Cohort, and an assessment of vICU coverage for the Cardiac ICU Cohort after 3 full years of operations.

## Profile and Special Needs of the Cardiac Icu Cohort

CICU and CVICU are specialized clinical settings with unique patient populations, as shown in Table 1.^9,10^ CICU beds are commonly occupied by patients with acute decompensated heart failure and those on inotropes or mechanical support devices awaiting heart transplant. Similarly, CVICU patients are typically those who have undergone cardiac surgery, aortic dissection repair, or other complex vascular procedures, and thoracic transplantation. Both units receive overflow patients with general medical, neurological, or surgical conditions.

**Table 1:** Cardiac Intensive Care Cohort patient population.


PATIENT POPULATION	DESCRIPTION

**Medicine, general surgery and neurology patients**	Respiratory failure secondary to pneumonia, status post complex abdominal surgery, cerebrovascular accident status post intervention.

**Preoperative cardiovascular, cardiac and vascular surgery patients**	Unstable patients being evaluated for aortic dissection repair or cardiac surgery with or without devices. Heart transplant patient on inotropes or with intra-aortic balloon pump (IABP) or Impella.

**Cardiovascular and thoracic surgery patients**	Coronary artery bypass, valve repair or replacement, cardiac tumor resection, pneumonectomy, lobectomy, esophagectomy, aortoesophageal repair.

**Complicated and combined vascular surgery patients**	Post left ventricular assist device, heart transplant patients, aortic dissection repair (endovascular or open), superior vena cava syndrome repair, heart and/or lung transplant or combined with liver or kidney transplant.

**Congestive heart failure patients**	Acute decompensated heart failure, including those with or without cardiac devices; any patient requiring invasive cardiac monitoring, status post percutaneous coronary intervention for ST elevation myocardial infarction.

**Extracorporeal membrane oxygenation (ECMO) patients**	Venovenous in cardiac intensive care unit and venoarterial in cardiovascular intensive care unit.

**Cardiogenic shock patients**	Patients in need for mechanical circulatory support such as IABP, Impella, durable ventricular assist device, or ECMO.


These patients are managed by a specialized team of clinicians with specific expertise in cardiac critical care and cardiovascular as well as thoracic surgical critical care.^11^ The Cardiac ICU Cohort intensivist team must be proficient in early recognition and management of cardiogenic shock, including various mechanical circulatory support devices as well as their indications, contraindications, and complications.^12,13^ The intensivist also must be adept at interpreting and managing arrhythmias, identifying signs of cardiac ischemia, and managing heart failure, among other diagnoses.^14,15^ The Advanced Practice Providers (APP) are nurse practitioners and physician assistants specially trained in managing cardiac and cardiovascular patients. Both ICUs have their own set of day and night intensivists and APPs.^16^

## The Virtual Intensive Care Unit Platform

**Figure 1:**
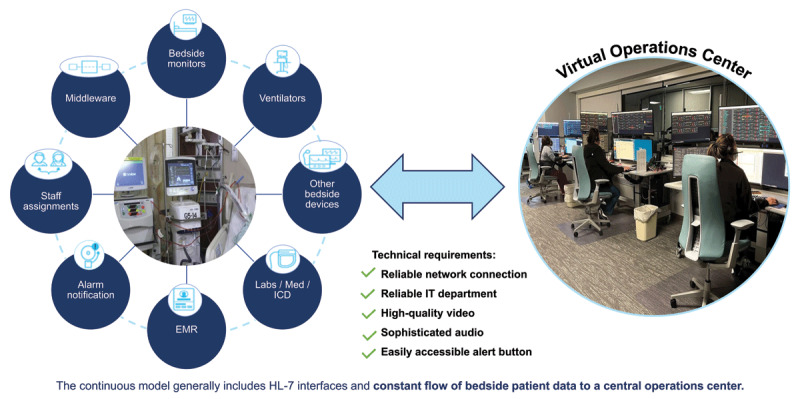
A primer on the virtual ICU (vICU) continuous model. The vICU platforms connect the hub (operations center) to the patient’s room using the audiovisual (AV) communication infrastructure, and patients are monitored by virtual intensivists and virtual critical care nurses vRNs) from the operations center using the AV infrastructure.^18,19,20,21,22,23,24^ HL-7: Health Care Level 7; EMR: electronic medical record; ICD: International Classification of Diseases

To understand the complexity in establishing a vICU, one must first understand the vICU platform (Figure 1). Virtual ICU platforms connect the hub (operations center) to the patient’s room using the audiovisual (AV) communication infrastructure. The vMDs and vRNs monitor the patients from the operations center using the AV infrastructure. The physical layout of the vICU should maximize communications between the teams while ensuring patient privacy.^17^ The highest fidelity video connection is important for neurologic and skin exam, drips, and ventilator settings, especially in low light conditions. A sophisticated audio connection enables multiple providers to communicate between the room and vICU. High quality microphones are essential during Code Blue events, denoting a situation where a patient requires immediate resuscitation, typically due to a cardiac or respiratory arrest. A properly positioned virtual alert button rapidly notifies the vICU of critical events. The data transfer and interface protocols used in the high-acuity, continuous-care vICU programs should meet or exceed the industry standard of Health Care Level 7. The operations center gathers “big data” and provides decision-support analytics for the clinicians. The enormous volume of incoming data necessitates the use of artificial intelligence (AI)-driven algorithms to curate abnormal patient physiology and human oversight to determine whether a patient is unstable. A multidisciplinary virtual team enhances the quality of the vICU-integrated critical care delivered to the patient bedside.^18,19,20,21,22,23,24^

## Launching a vICU in Houston Methodist’s Cardiac ICU Cohort

HMH has historically favored a staffing model that provides 24/7 bedside coverage by critical care intensivists in all ICUs, including its CICU and CVICU.^25^ However, this model presented staffing challenges, requiring almost daily locums staffing in the CICU and extra shifts by in-house intensivists. A feasibility assessment conducted in 2018 indicated that a vICU program would improve staffing efficiency by providing continuous access to critical care specialists while lowering staff burnout for bedside clinicians. The vICU solution would have reduced night staffing from two in-person intensivists to one support intensivist (an in-person cardiac intensivist) covering the 66-bed Cardiac ICU Cohort, supported by the vICU team.

In addition to improving staffing efficiencies, tele-critical care programs offer several other key benefits, including (1) improved coordination of care, (2) increased night-time coverage, and (3) Clinical Decision Support Systems, allowing enhanced monitoring and early identification of patient deterioration.^26^ With proper implementation, these programs have shown potential survival and quality benefits in the appropriate settings.^18,23^ Moreover, the assessment suggested that HM’s vICU program would provide a range of unique services to the ICUs such as “teleproctoring” of the newer nurses at the ICU bedside.^27^

Teleproctoring entails the oversight of newer bedside clinicians by more experienced physicians and nurses working in a vICU.^28^ Given the high turnover of RNs at the bedside,^29^ the experience of the vICU to provide a second set of eyes—not only on the patients but also on the nursing staff—offered valuable support. The ability to conduct skills check-off, auditing, infection control rounds, and training were potential areas of support to newer staff and relief for more senior bedside RNs whom others rely on for proctoring and support. While much of the focus had been on nursing, staff in other disciplines such as respiratory care, physical therapy/occupational therapy, and pharmacy also stood to benefit from efficiency gains by leveraging the nightly vICU resources.

Despite general support for the vICU program, the contemplated shift in staffing resources from bedside to virtual care raised concerns about the maintenance of quality and safety due to the high acuity and complexity of the Cardiac ICU Cohort patients. Many stakeholders, including the CV critical care team, cardiologists, and cardiovascular surgeons, were reluctant to move away from the in-person staffing, a resource allocation shift that was essential to support the vICU implementation. Ultimately, the need to create a sustainable model in physician staffing overshadowed initial concerns for the degradation of quality. These concerns were mitigated as the vICU added staff with specific training in managing cardiac and cardiovascular ICU patients and as the longitudinal outcomes became more tangible.

## Planning for the Launch

The launch of the vICU program in the winter of 2019 to 2020 was preceded by many years of strategic planning, including consultations with other US hospital systems that had successfully implemented their own tele-critical care programs. In 2019, over a period of a few months, HMH built the necessary infrastructure, including the installation of the audiovisual hardware at the bedside, and constructed the vICU Operations Center, underscoring HMH’s significant financial commitment.^30^

Among the many prerequisites for vICU “going live” for night coverage, three steps were critical for a successful launch: (1) formation of an experienced vICU clinical team using in-house critical care experts and/or third-party vendors; (2) consultations with clinicians to understand and account for their concerns and to secure a buy-in on the merits of the vICU program; and (3) co-creation of new workflows to weave together the functions of vICU staff and bedside clinicians without disrupting the existing workflows.

As vICU was going live in early 2020, COVID-19-related census and patient acuity increased significantly in the Cardiac ICU Cohort, with more patients on extracorporeal membrane oxygenation (ECMO) than ever before.^31^ Recognizing the importance of a systems approach to meet the stakeholders’ needs and expectations for a successful adoption of vICU and to address the related quality-of-care concerns, we devised a stepwise approach to integrate vICU into the Cardiac ICU Cohort that emphasized development of trust and confidence between bedside and virtual teams.^32,33^

## Stepwise Integration

Given their specialized patient population, the Cardiac ICU Cohort was introduced to the nocturnal vICU services in six discrete steps: (1) introduction of proprietary algorithms that form the core of vICU’s monitoring platform; (2) recruitment of qualified vICU staff with expertise in managing cardiac and cardiovascular patients; (3) hosting a series of “meet-and-greet” events between bedside and virtual teams outside clinical settings as well as reciprocal tours of ICUs and the vICU operations center; (4) “soft” launch of vICU without dismantling the status quo intensivist model but encouraging the bedside staff to utilize vICU services whenever possible; gradually, regular mandatory nocturnal rounds were introduced with the bedside intensivists and APPs and the vICU staff to promote confidence-building interactions; (5) select admissions assigned to be performed by the bedside staff and the vMD, with the bedside intensivist available as needed; and (6) reduction of nightly MD staffing with greater reliance on vICU staff and the use of a support intensivist who floats between the CICU and the CVICU for procedures, codes, family meetings, and other bedside activities requiring the physical presence of an intensivist.

**Figure 2:**
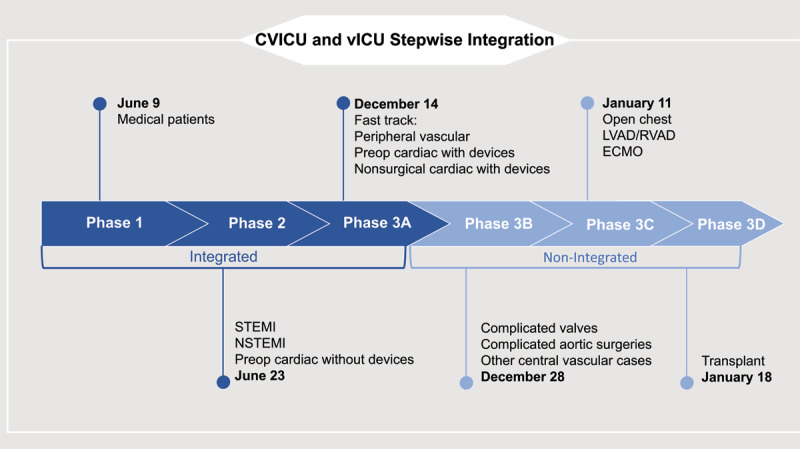
The introduction of virtual intensive care unit (vICU) services to the cardiovascular ICU required stepwise integration, taking almost 1 year. Two months after the initial launch, the CVICU patient population was divided into integrated and nonintegrated groups. STEMI: ST elevation myocardial infarction; NSTEMI: non-ST elevation myocardial infarction; LVAD: left ventricular assist device; RVAD: right ventricular assist device; ECMO: extracorporeal membrane oxygenation

The stepwise integration in the CVICU posed another challenge: a subset of this unit’s high-acuity cardiac patients with complicated surgeries or requiring cardiac devices necessitated hands-on management by in-person clinicians. This group was designated as the “non-integrated” cohort (Figure 2).

Throughout the post-integration year, continuous assessment was performed by eliciting feedback from the APP’s, nursing, support intensivists, cardiologists, and cardiovascular surgeons. This feedback informed the weekly discussion between the medical directors of the ICUs and vICU to assess and improve the service line communication.

## Participatory Development of New Workflows

**Figure 3:**
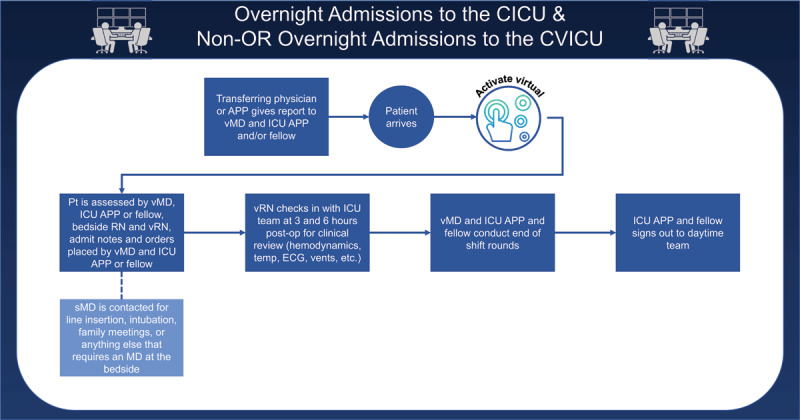
Workflow describing overnight integrated patient admissions to the CICU and nonoperating room (non-OR) patients to the CVICU, with the roles and responsibilities of the virtual MD (vMD). CICU: cardiac intensive care unit; CVICU: cardiovascular ICU; sMD: support intensivist; APPs: advanced practice providers; RNs: registered nurses; vRNs: virtual RNs; ECG: electrocardiogram

**Figure 4:**
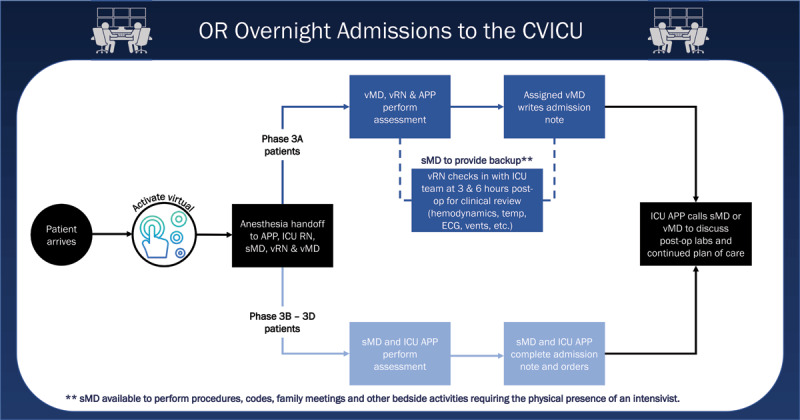
Workflow describing overnight admissions of patients from the operating room (OR) to the CVICU, with the roles and responsibilities of the virtual MD (vMD) covering the integrated patients, support intensivist (sMD) covering the nonintegrated patients, advanced practice providers (APPs), registered nurses (RNs) and virtual RNs (vRNs). CVICU: cardiovascular intensive care unit; ECG: electrocardiography

A participatory design approach was used to elicit feedback from vICU and CICU clinicians to create new “best-practice” workflows that detailed the specific roles of virtual and bedside teams (Figure 3). More nuanced workflows were created for cardiac and vascular surgery patients requiring specialized medical devices (Figure 4). All the integrated workflows were designed to complement bedside protocols for procedures and specific care while providing a second set of eyes on potential patient deterioration.

During the early integration phases, the new workflows and the roles of vICU staff were continuously reassessed and adjusted as needed to address any perceived or real concerns for patient safety. As part of the ongoing reassessment, weekly meetings were scheduled to consider which complex operating room cases would benefit from hands-on management by the support intensivist. This iterative approach allowed us to balance the support intensivist and vMD workloads and optimize the workflows in the Cardiac ICU Cohort.

## New Staffing Alignment

**Figure 5:**
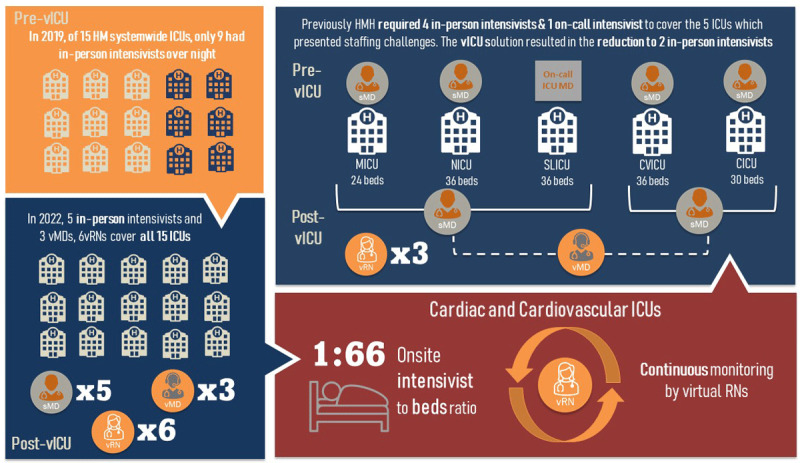
In-person intensivist staffing alignment pre-vICU and post-vICU across the entire Houston Methodist system, including HM hospital’s CICU and CVICU. CICU: cardiac intensive care unit; CVICU: cardiovascular ICU; vMD: virtual MD; sMD: support intensivist; vRN: virtual RNs; MICU: medical ICU; NICU: neonatal ICU: SLICU: surgical ICU

For post-launch staffing alignment, we kept two experienced specialty APPs/fellows at the bedside during the night in each ICU as the first-line critical care providers, who reported to either vMD or support intensivist. Under this staffing arrangement, only one support intensivist was assigned to cover the Cardiac ICU Cohort. With the addition of vICU support, HM was able to cover all 15 of its systemwide ICUs using five in-person intensivists and three vMDs at night. At HMH, the vICU support resulted in the reduction of nightly in-person coverage by two intensivists as shown in Figure 5. While CICU became fully integrated, the subset of non-integrated patients (5-20 patients per night) in the CVICU were managed by the bedside team. However, if any patient required point-of-care ultrasound (POCUS), chest tube insertion, or other complex bedside procedures, the support intensivist could be called to the bedside for assistance. If the support intensivist was busy elsewhere, the bedside team could access the vMD even for the nonintegrated patients.

At night, all HM ICUs, including the Cardiac ICU Cohort patients, were continuously monitored by virtual RNs from the operations center. In early 2022, a daytime episodic vRN model was created to allow remote monitoring and support of patients in the Cardiac ICU Cohort while the bedside nurse was out of the unit on transport with another patient.

## Impact of vICU on CICU and CVICU

Anecdotal evidence from continuous assessments suggests that the integration of vICU has resulted in an increase in intensivist satisfaction with their work. Prior to integration, two physicians were required to be in-house every night to cover all the patients in the Cardiac ICU Cohort. Post-integration of the vICU, only one physician is now required to be in-house at night as the support intensivist, cutting each intensivist’s night-call workload from six to three nights a month. With the efflux of experienced nursing staff during this period, the use of experienced nurses as vRNs allowed them to teleproctor the less experienced nurses. Similarly, the vMDs provided oversight for the newer APPs.

## Specialists’ Enhanced access to Patients: Consultant Bridge

The vICU command center also enabled specialists or consultants to connect to a patient’s ICU bed from any remote location at all hours using a secure video conferencing link and an application called “consultant bridge.” Consultant bridge has been used to connect cardiothoracic, transplant, and vascular surgeons with their respective patients in case of emergent need. The consultant bridge is also used by CV surgeons in several HM hospitals to remotely visualize, evaluate, and communicate plans of care with both bedside and vICU teams.^34^

Cardiologists are expected to use the consultant bridge to evaluate patients at the regional hospitals for progressive cardiogenic shock in order to initiate timely transfer to HMH for advanced heart failure therapies. The CICU intensivists can also use the consultant bridge to conduct preliminary ECMO evaluations of patients at regional hospitals to assess their potential transfer to the main campus. If the transfer is not possible, the CICU intensivists can use the consultant bridge to co-manage the ECMO patients with the intensivists in the HM regional hospitals.

## APACHE-IV Remote and Continuous Monitoring

**Figure 6:**
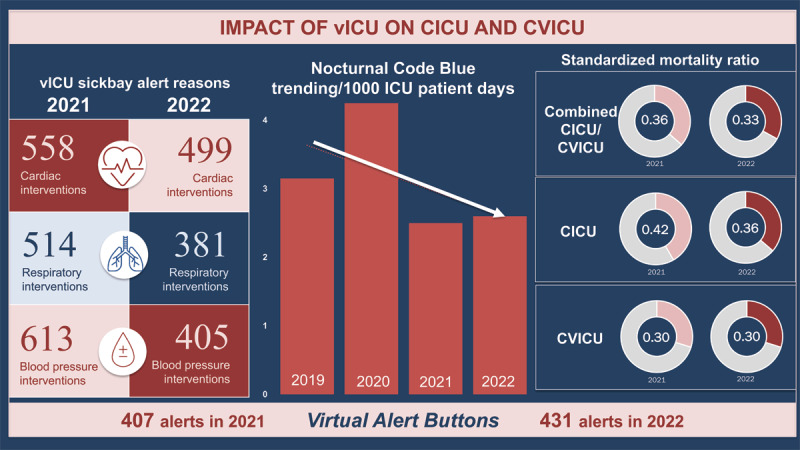
Number of cardiac, blood pressure, or respiratory alerts generated during the monitoring hours (2021–2022); Virtual alert triggered by bedside providers; nocturnal Code Blue trending for 1,000 patient days (2019–2022); and the Standardized Mortality Ratio for the Cardiac ICU Cohort (2021 & 2022). vICU: virtual intensive care unit; CICU: cardiac ICU: CVICU: cardiovascular ICU

Integrated into all the bedside monitoring systems, SickBay^TM^ (Medical Informatics Corporation), the vICU monitoring platform, acts as the automated sensory limb of the vICU augmented intelligence. The system generates alerts based on vital sign instability measured by the bedside monitors and other interfaced devices or data from the electronic medial record flowsheet.^35,36^ In the period between 2021 and 2022, 2,970 alerts (cardiac, blood pressure, or respiratory) were generated during the monitoring hours. In addition, bedside providers actively triggered an additional 838 virtual alerts (Figure 6). A trend in the reduction of Code Blue events was noted in the nocturnal hours from 2019 to 2022 for the Cardiac ICU Cohort (Figure 6). When comparing 24-hour periods in 2019/2020 to 2021/2022, a significant reduction in the rate of Code Blue events was noted (Rate Ratio 0.82; 0.68-0.96, 95% CI).

The vICU functions not only as a care support mechanism but also a robust data repository capable of providing global ICU benchmarking data. A commonly used severity of illness scoring system, known as The Acute Physiology And Chronic Health Evaluation (APACHE-IV), is used to provide ongoing risk-adjusted outcomes.^37^ This tool gives a robust assessment of individual ICU performance with regard to mortality and length of stay relative to peers. The overarching goal of the vICU implementation was to deliver the same high quality patient care more efficiently. The Standardized Mortality Ratio (SMR)^38^ for the Cardiac ICU Cohort in 2021 and 2022 was 0.36 and 0.33, respectively (Figure 6). This represents a very low risk-adjusted ICU mortality, in line with the goals of maintaining quality while improving efficiency.

## Discussion: Barriers and Opportunities

The long-standing shortage in healthcare staffing, the demands of a global pandemic, and the enabling forces of technology formed the backdrop of Houston Methodist’s plan, launch, and integration of its virtual ICU program. The vICU program augmented bedside patient care with additional virtual staff without compromising the quality of patient care while decreasing the workload of our ICU staff. The specialized needs of a complex Cardiac ICU Cohort were identified and met over the launch and integration process. The successes of the vICU program have validated our institution’s firm commitment to quality and patient safety while enhancing the efficiency and utility of our valuable resources. Even the relative shortcomings of our vICU program have proven to be instructive.

Despite multiple years of preparation, communication, and discussion, the implementation of the vICU may have been better primarily in the area of change management. The addition of intensivists with cardiovascular critical care expertise to the vICU staff addressed an important gap and bolstered the lines of communication between bedside and the vICU. The elevation of CICU and CVICU directors as the primary decision-makers for the in-house cardiologists and CV surgeons further streamlined the communication process without marginalizing anyone’s opinion. In addition, the initial rollout did not include daily or weekly discussions between these teams, which were added later to improve trust and utilization of specific cardiac workflows. The decision to exclude a selected group of complex postoperative CVICU patients from vICU coverage proved to be effective as these patients required in-person support and coordination.

At the outset, the vICU service line was not integrated with the ICU quality structure—an oversight that was corrected by adding the medical director of the vICU to the ICU quality and safety committee for the purpose of reporting and tracking performance improvement.

For the first 3 years of the vICU operations, the staffing teams were strictly segregated between the bedside clinicians (both RNs and MDs) and vICU staff. Now, bedside clinicians have opted to do shifts as vMDs and vRNs, a trend that is fostering improved communication and trust among providers. Given a burgeoning patient population with increasing Case Mix Index, Houston Methodist’s experiment and adoption of vICU has yielded quantifiable improvement in work conditions marked by an increased number of providers during the day and greater trust and confidence among them.

## Limitations

Since critical care delivery cannot be wholly virtual, vICUs function to augment, not replace, traditional bedside care. Patients still maintain regular interactions with bedside nurses, advanced practice providers, residents, fellows, and support intensivists. Although the technological advances in vICUs have yielded exceptional audiovisual quality, providers are not able to physically touch the patient, making physical exam less optimal. To overcome these challenges, vICU providers must become adept at detecting visual and auditory cues during a patient encounter. Increasingly, bedside providers are using POCUS and, by extension, tele-POCUS to perform focused critical care echocardiograms and look for signs of pneumothorax, ascites, and consolidation.^39,40^ Additionally, ventilator waveforms are a tremendous resource in better understanding outflow obstruction, air leaks, high pressures and changes in patient’s lung compliance.

Furthermore, measurement of the vICU’s impact can be limited by their quasi-experimental designs, such as pre-post and difference-in-differences analyses, due to the inherent difficulties of blinding a vICU study. Limitations within the technology can become apparent with occasional connection outages, impaired sound quality, internet speeds and camera quality. These issues are mitigated by careful planning for downtime occurrences and contingencies in the event of a technological failure.

Finally, successful implementation of a vICU program requires full buy-in by all the stakeholders. This involves managing expectations, constantly adjusting workflows, providing ongoing education and reorientation for new staff. The organizational structure also needs continuous modification to ensure timely, consistent, and integrated rather than parallel or siloed patient care.

## Brave New World of Cardiac ICUs

The vICU operations have expanded the boundaries of traditional ICUs by allowing critical care specialists to remotely manage ICU patients using technological hardware, big data, and machine learning. By contrast, the brick-and-mortar Cardiac ICU Cohort provides essential patient-specific, patient-centric, hands-on care that does not afford the luxury of examining large sets of data in real-time for the benefit of a single patient. To date, we have barely scratched the surface of advances in patient care that are possible in the brave new world of cardiac ICUs.

Now, with multiple years of continuous data and utilization of algorithms as well as physician comfort in the basic uses, research scientists and clinicians have begun to study the potential of AI to predict, track, and trend patients. The current teams have the capacity to integrate emerging technologies into new workflows and protocols to improve ICU patient care. The opportunity to create a more multidisciplinary vICU team with APPs, pharmacists, respiratory therapists, social workers, and infection control nurses can help to drive more efficient and effective ICUs.

Tele-critical care requires an infrastructure capable of sensing, synthesizing, and then acting upon the immense data flow from the bedside.^41,42,43^ With its unique design, HM’s vICU can effectively navigate patient environments in the age of big data. The algorithms underlying our vICU analytics continue to be improved and grow with the needs of our ICU patient population. For example, the heart transplant team reported that patients in cardiogenic shock are transferred from HM’s regional hospitals to HMH’s advanced heart failure team and CICU later than necessary for optimal management. With advances in AI, algorithms that proactively identify those patients who are ideal for transfer earlier on are now well within our reach.

We foresee several areas where AI will provide impactful benefits in the not-too-distant future. AI will enable us to (1) differentiate congestive heart failure from other causes of lung disease and to quantify the amount of pulmonary edema secondary to it, using process imaging data; (2) identify left ventricular systolic dysfunction using AI-electrocardiogram and reduce mortality in CICU patients and (3) identify disease phenotypes or endotypes, which can inform personalized management and clinical trials.^44,45^ These AI-research driven advances, in addition to many others, herald a new age of patient care in the ICUs.

## Key Points

- Houston Methodist’s need to support a 24/7 staffing model to serve all its intensive care units (ICUs), including the cardiac and cardiovascular ICUs, provided the strategic rationale for adopting a tele-critical care program.
- The Cardiac ICU Cohort served cardiac, cardiovascular, and thoracic patients with highly complex and specialized needs, requiring specialty trained virtual ICU (vICU) staff with experience in treating such patients.
- Ongoing consultations with—and buy-in from—key stakeholders, including cardiologists and cardiovascular surgeons, were essential for the successful launch and integration of the vICU into the Cardiac ICU Cohort.
- The integrated vICU and Cardiac ICU Cohort platform has yielded promising results, reducing the nocturnal workload of in-house staff by 50% while delivering a systemic reduction in Code Blue events. Additionally, the program has resulted in maintenance of and slight reduction in risk-adjusted mortality.
- The vICU platform enables the use of big data and artificial intelligence to predict adverse events before they occur in the Cardiac ICU Cohort. The vICU technology holds great promise to improve patient outcomes.
